# Tumor-infiltrating mast cells predict prognosis and gemcitabine-based adjuvant chemotherapeutic benefit in biliary tract cancer patients

**DOI:** 10.1186/s12885-018-4220-1

**Published:** 2018-03-21

**Authors:** Xiaobo Bo, Jie Wang, Tao Suo, Xiaoling Ni, Han Liu, Sheng Shen, Min Li, Yueqi Wang, Houbao Liu, Jiejie Xu

**Affiliations:** 1 Department of General Surgery, Zhongshan Hospital, Fudan University, Shanghai, 200032 China; 2 Department of Biochemistry and Molecular Biology, School of Basic Medical Sciences, Fudan University, Shanghai, 200032 China

**Keywords:** Mast cells, Biliary tract cancer, Surgery, Overall survival, Adjuvant chemotherapy

## Abstract

**Background:**

Recent studies have reported TIMs play an important role in tumors progression or regression, but the effect of TIMs in biliary tract cancer remains unclear. The aim of this study is to investigate the prognostic value of tumor infiltrating mast cells (TIMs) and its influence on gemcitabine-based adjuvant chemotherapy (ACT) benefits in biliary tract cancer patients after surgery.

**Methods:**

TIMs were evaluated by immunohistochemical staining of tryptase in 250 patients with resected gallbladder carcinoma (GBC) or extrahepatic bile duct carcinoma (EBDC) from Zhongshan Hospital. The relationships between TIMs and clinicopathological factors and postoperative prognosis were analyzed respectively.

**Results:**

High TIMs infiltration was significantly correlated with prolonged overall survival (OS). Furthermore, multivariate analysis indicated TNM stage and TIMs as independent prognostic factors for OS. Patients with high TIMs infiltration appeared to significantly benefit from Gemcitabine-based ACT in the discovery and validation cohorts. Spearman analysis identified that TIMs infiltration were positively correlated with anti-tumor CD8+ T cells.

**Conclusion:**

TIMs infiltration is an independent favorable prognostic factor in GBC and EBDC patients, which could better stratify patients with different prognosis and predict benefit from gemcitabine-based ACT.

**Electronic supplementary material:**

The online version of this article (10.1186/s12885-018-4220-1) contains supplementary material, which is available to authorized users.

## Background

Biliary tract cancer (BTC), arised from the epithelial cells of the biliary tract, encompassing malignant neoplasms of gallbladder and bile duct [1]. BTC comprises about 3% of gastrointestinal tumors and the incidence of it seems to be increasing [2]. Although a number of risk factors and molecular mechanism for BTC have been found, the 5-year survival is still as low as 10% [2]. Gallbladder cancer (GBC) is the most common biliary tract malignancy and the seventh most common gastrointestinal cancer, with high mortality rate owing to the early infiltration of the tumor cells into the liver by lymphatic, perineural and hematogenous routes [3]. Extrahepatic bile duct cancer (EBDC) consists of hilar and distal bile duct carcinoma, with a poor 5-year survival rate ranging from 20%–45% [4]. In spite of GBC and EBDC originate in different anatomic biliary tree, there are still overlapping etiologic risk factors, patients characteristics, histopathology and molecular biology. Complete resection is the only potentially curative therapy for patients with biliary tract cancer; however, biliary tract tumors are usually asymptomatic at early stage and the majority of patients present with advanced, unresectable tumors [5]. Therefore, surgery alone is not sufficient treatment for biliary carcinoma, especially in advanced stages. And the adjuvant therapeutic modalities, such as chemotherapy or radiotherapy, are needed to improve long-term survival [6].

Although recent BILCAP study have reported capecitabine could extend survival in UK patient, there is still no established adjuvant chemotherapy other than several attempts to identify effective agents for patients with biliary cancer in Asia [6]. According to NCCN clinical practice guideline of hepatobiliary cancers, gemcitabine-based adjuvant chemotherapy is recommended for biliary tract cancer patients. However, no prospective studies have demonstrated the efficacy of adjuvant gemcitabine for patients with biliary carcinoma [7]. Therefore, there is an urgent need for identifying a precise biomarker to better predict patient benefitting from adjuvant chemotherapy.

Tumors contain cancer cells and recruit normal cells, creating the tumor microenvironment, including innate immune cells (neutrophils, macrophages, dendritic cells, natural killer lymphocytes, mast cells) and adaptive immune cells (T and B lymphocytes) [8]. Both innate and adaptive immune systems have been implicated in promoting and preventing tumor growth. TIMs are attracted by tumor microenvironment by stem cell factor (SCF) secreted by tumor cells and secrete several angiogenic factor as well as matrix metalloproteinases (MMPs), which promote tumor vascularization and invasiveness, respectively [9, 10]. On the contrary, TIMs exert inhibition of tumor cell growth through releasing IL-1, IL-4, IL-6, IL-8, monocyte chemotactic protein-3 and -4 (MCP-3 and MCP-4), transforming growth factor beta (TGF-β), and chymase [10]. TIMs in prostate cancer, [11] metastatic bladder cancer, [12] Hodgkin’s lymphoma, [13] thyroid cancer, [14] pancreatic cancer, [15–17] metastatic colorectal cancer, [18] and non-small cell lung cancer [19] were associated with poor prognostic outcome,whereas TIMs in local colon cancer, nonmetastatic and invasive breast cancer confer a favorable prognosis [20, 21]. A recent research reported TIMs participate in the progression and metastatic potential of cholangiocarcinoma [22]. However, the effect of peritumoral TIMs for biliary tract cancer and the precise function mechanisms in tumor progression still remain obscure. Furthermore, few studies have demonstrated a relationship between TIMs and clinical outcomes of patients with biliary tract cancer.

In this study, we explored whether TIMs could predict the survival of GBC and EBDC patients who received gemcitabine-based chemotherapy after operation by immunohistochemical staining and evaluated its correlation with clinicopathological characteristics.

## Methods

### Patients selection

A total of 250 consecutive patients with GBC or EBDC, including 164 with GBC, 64 with perihilar cholangiocarcinoma (PHC) and 22 with distal cholangiocarcinoma (DC) underwent surgical resection between May 2004 and April 2012 at Zhongshan Hospital, Fudan University (Shanghai, China) were recruited in present study. Ampullary tumors were excluded from the EBDC due to potential different biology. The total 250 patients were assigned into two independent patient cohorts: discovery set (*n* = 115) and validation set (*n* = 135) according to different follow-up time. Of these patients, 219 received radical resection (R0), 100 received gemcitabine-based chemotherapy after surgical resection (at least one cycle). This study was approved by the Ethics Committee of Zhongshan Hospital, and written informed consent was obtained from all patients. The clinicopathological and baseline demographic characteristics of the patients including age, gender, tumor location (PHC, DC or GBC), tumor differentiation, vascular invasion and TNM stage were collected retrospectively. OS was calculated from the date of surgery to the date of death or censored at the date of last visit. The median follow-up time of the patients after operation was 26.7 months, 11.7 months for discovery set and validation set, respectively.

### Tissue microarray and immunohistochemistry

Tissue microarray (TMA) was established with formalin-fixed paraffin-embedded surgical specimens, and immunohistochemical staining was performed on TMA according to the protocols previously described [23] with appropriate antibodies after control staining (Mouse anti-tryptase monoclonal antibody, diluted 1:1000; Abcam, Cambridge, UK; anti-CD8, IR623, DAKO, ready-to-use). The negative control sections were treated equally with primary antibody omitted. The number of MCs and CD8 + T cells per field was evaluated with Image pro plus 6.0 (Media Cybernetics Inc., Bethesda, MD). Identical settings were used for each photograph. Positive staining were calculated under high magnification field (HPF, 400×). The intensity of MCs or CD8 + T cells was scored as the mean number of MC or CD8 + T cell positive/HPF from entire filed independently by two pathologists. The cut-off point for the high/low MCs or CD8 + T cells infiltration was determined with the X-tile software. Overall survival curves were plotted by the Kaplan-Meier method, and log-rank test was used to analyze the difference between subgroups.

### Statistical analyses

Statistical analysis was performed with SPSS 22.0 (IBM Corporation, Armonk, NY, USA), Medcalc Software (version 15.2.2; Medcalc, Mariakerke, Belgium) and Stata SE, version 13.0 (Stata, College Station, TX). Chi-square test or Fisher exact test was used to evaluate the correlation between clinicopathological features and immunohistochemical variables. Continuous variables were analyzed by means of *t* test. Univariate and multivariate regression analysis was performed with Cox proportional hazards regression model. To further explore the prognostic value between TIMs density and clinical outcomes, we applied Kaplan-Meier analysis to compare overall survival (OS) between subgroups. The log-rank test was used to compare survival rates. All tests were two-sided, and *P* < 0.05 was regarded as statistically significant.

## Results

### Immunohistochemical findings

**Fig. 1:**
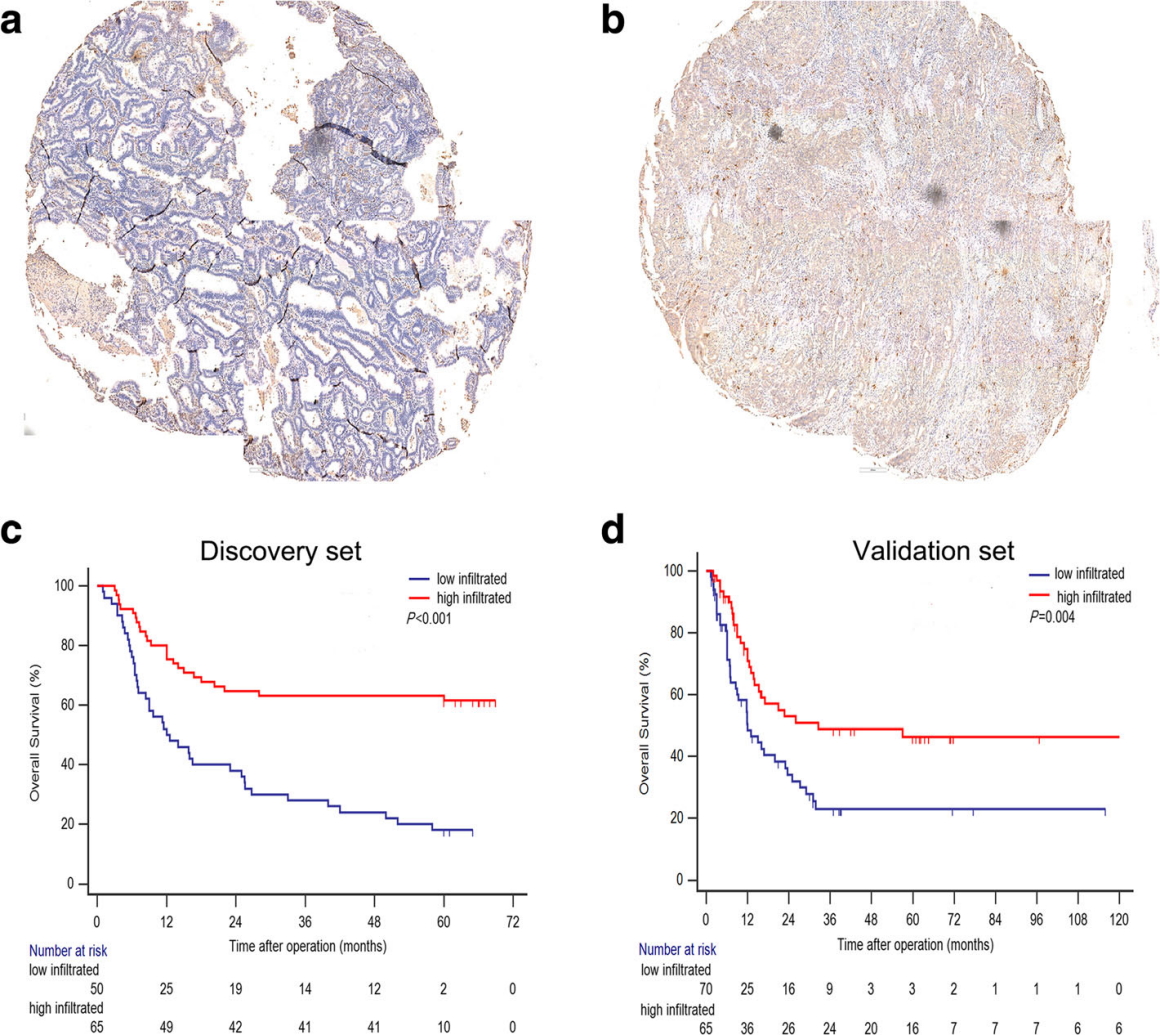
Representative images of tumor infiltrated mast cells (TIMs) staining in BTC. (**a**) Low TIMs infiltration. (**b**) High TIMs infiltration. Scale bar: 200 μm (original magnification × 400). (**c**, **d**) Kaplan-Meier analysis of overall survival in the discovery set and validation set. *P* value was calculated by log-rank test

The tryptase positive staining represented mast cells were detected located in the tumor tissues in a diffused manner (Fig. 1a, b). The infiltration of TIMs in sequential slides per specimen were found the varied largely. The density of TIMs infiltrated tissues ranged from 0 to 112 cells/HPF, and 0 to 118/HPF in the discovery set and validation set. The median value (17/HPF) derived from the discovery set was defined as cut-off for high and low mast cells infiltration, and applied to the validation set. Patients with high TIMs infiltration had obviously better OS than those with low TIMs infiltration in the discovery set and validation set, respectively (Fig. 1c; d).

### Relationships between TIMs infiltration and baseline characteristics

Baseline characteristics of the 250 patients are described in Table 1. No factors were associated with TIMs infiltration in both the discovery set and validation set. In the discovery set, TIMs infiltration was negatively correlated with the lymph node metastasis (*P* = 0.008). while in the validation set, TIMs infiltration was positively associated with vascular invasion in the validation set (*P* = 0.035). These heterogeneities can guarantee the predictor has universal application across heterogeneous population of patients in different regions. No correlations between TIMs infiltration and other clinicopathological factors was observed. The associations between CD8+ T cells infiltration and baseline characteristics were list in Additional file 1: Table S1.

**Table 1:** Association between mast cell infiltration and patient characteristics

			Discovery set (*n* = 115)					Validation set (n = 135)		
Characteristic	Patients		Mast cell infiltration			Patients		Mast cell infiltration		
	Number	%	Low (*n* = 50)	High (n = 65)	*P* ^a^	Number	%	Low (*n* = 70)	High (*n* = 65)	*P* ^a^
Age, years					0.71					0.61
Mean ± SD^b^	62.56 ± 10.26		62.16 ± 11.51	62.86 ± 9.26		63.67 ± 11.62		61.56 ± 11.72	66.18 ± 10.94	
Gender					0.69					0.30
Female	62	53.91	28	34		91	67.41	50	41	
Male	53	46.09	22	31		44	32.59	20	24	
Tumor location					0.31					0.26
Perihilar	40	10.43	19	21		24	17.78	14	10	
Distal	12	63	7	5		10	7.41	3	7	
Gallbladder	63	54.78	24	39		101	74.81	57	44	
T-stage					0.25					0.91
T1–2	41	35.65	17	24		16	11.85	9	7	
T3	44	38.26	16	28		79	58.52	41	38	
T4	30	26.09	17	13		40	29.63	20	20	
N-stage					0.008					0.87
N0	62	53.91	20	42		114	84.44	59	55	
N1,2	53	46.09	30	23		21	15.56	11	10	
TNM stage					0.12					0.93
I-II	39	33.91	16	23		16	11.85	9	7	
III	36	31.30	11	27		74	54.81	38	36	
IV	40	34.79	23	17		45	33.34	23	22	
Differentiation					0.98					0.21
Well, Moderate	76	66.09	33	43		59	43.70	27	32	
Poor	39	33.91	17	22		76	56.30	43	33	
Residual tumor					0.16					0.21
R0	100	86.96	41	59		119	88.15	65	57	
R1	15	13.04	9	6		16	11.85	5	9	
Vascular invasion					0.49					0.035
Absent	64	55.65	24	27		106	78.52	60	46	
Present	51	44.35	26	38		29	21.48	10	19	
ACT					0.78					0.49
Absent	65	56.52	29	36		85	62.96	46	39	
Present	50	43.48	21	29		50	37.04	24	26	

*SD* standard deviation; ACT = adjuvant chemotherapy

^a^, *P* < 0.05 is considered statistically significant

^b^, The results of continuous variables are presented as mean ± SD (standard deviation)

### Univariate and multivariate regression analysis in patients with BTC

Univariate analysis was performed to identify the clinical significance of TIMs that might influence survival in the study. It was shown that T-stage, TNM stage, TIMs were significantly associated with OS in all these sets (Table 2, all *P* < 0.05). There was no relationship between TIMs and tumor location in multivaratie analysis. Cox regression analyses revealed that TNM stage (*P* < 0.001; *P* = 0.046) and TIMs (*P* < 0.001, 3.210(1.834–5.620); *P* = 0.002, 2.338(1.27–3.986)) were identified as independent prognostic factors in the discovery and validation set respectively (Table 2). CD8 + T cells infiltration is not an independent prognostic factors both in the discovery set (*P* = 0.602, 1.150(0.068–1.943)) and validation set (*P* = 0.218, 1.382(0.826–2.313)).

**Table 2:** Univariate and Multivariate Cox regression analysis of Overall survival

		Discovery set			Validation set	
Characteristic	Univariate analysis *P*^a^	Multivariate analysis		Univariate analysis *P*^a^	Multivariate analysis	
		Hazard Ratio (95% CI)	*P* ^a^		Hazard Ratio (95% CI)	*P* ^a^
Age at surgery, years	0.17			0.82		
Gender	0.18			0.42		
Female						
Male						
Tumor location	< 0.001		0.175	0.48		
Perihilar		Reference				
Distal		1.696(0.962–2.991)				
Gallbladder		1.150(0.680–1.940)				
T-stage	< 0.001			0.02		
T2						
T3						
T4						
N-stage	< 0.001			0.92		
N0						
N1						
TNM stage	< 0.001		< 0.001	0.02		0.046
II		Reference			Reference	
III		2.84(1.187–6.823)	0.019		2.317(0.860–6.243)	0.096
IV		4.694(2.064–10.677)	< 0.001		3.690(1.245–10.933)	0.018
Differentiation	0.15			0.005		0.30
Well-moderate					Reference	
Poor					1.346(0.767–2.360)	
Residual tumor	0.64			0.67		
R0						
R1						
Vascular invasion	0.18			0.94		
Absent						
Present						
TIMs	< 0.001		< 0.001	< 0.001		0.002
Low		Reference			Reference	
High		3.210(1.834–5.620)			2.338(1.371–3.986)	
CD8 + Tcells						
Low	0.023	Reference	0.602	0.027	Reference	0.218
High		1.150(0.068–1.943)			1.382(0.826–2.313)	

*CI* confidence interval, *TIMs* tumor infiltrating mast cells

^a^, *P* < 0.05 is considered statistically significant

### Correlations between TIMs infiltration and postoperative chemotherapy (ACT)

**Fig. 2:**
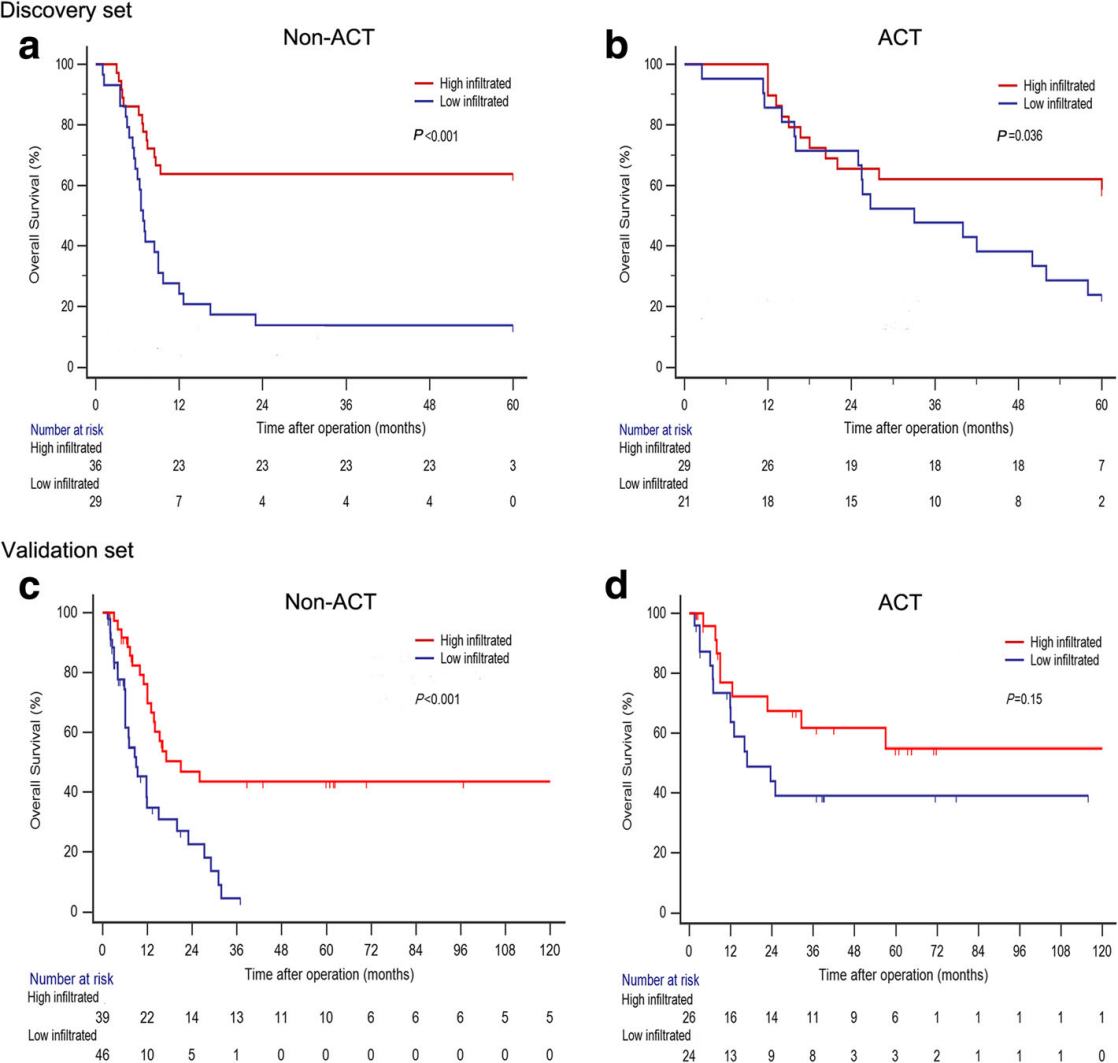
Association between TIMs infiltration and benefit from gemcitabine-based adjuvant chemotherapy (ACT). (**a**) Patients without ACT in the discovery set. (**b**) Patients with ACT in the discovery set. (**c**) Patients without ACT in the validation set. (**d**) Patients with ACT in the validation set. *P* value was calculated by log-rank test

Previous researches have reported that TIMs might kill tumor cells and enhance the effect of chemotherapy [24, 25]. Therefore, we estimated the benefit of gemcitabine-based chemotherapy according to the level of TIMs in patients who received adjuvant chemotherapy. As shown in Fig. 2, for patients without ACT treatment, the levels of TIMs infiltration was significantly correlated with OS (*P* < 0.001, *P* < 0.001) in the discovery and validation set. In addition, high TIMs subgroup could significantly benefit from ACT than low TIMs subgroup in the discovery set (*P* = 0.036). However, the association between TIMs and OS was not significant in patients with ACT in the validation set (*P* = 0.15).

### Relationships of TIMs and CD8+ T cells

**Fig. 3:**
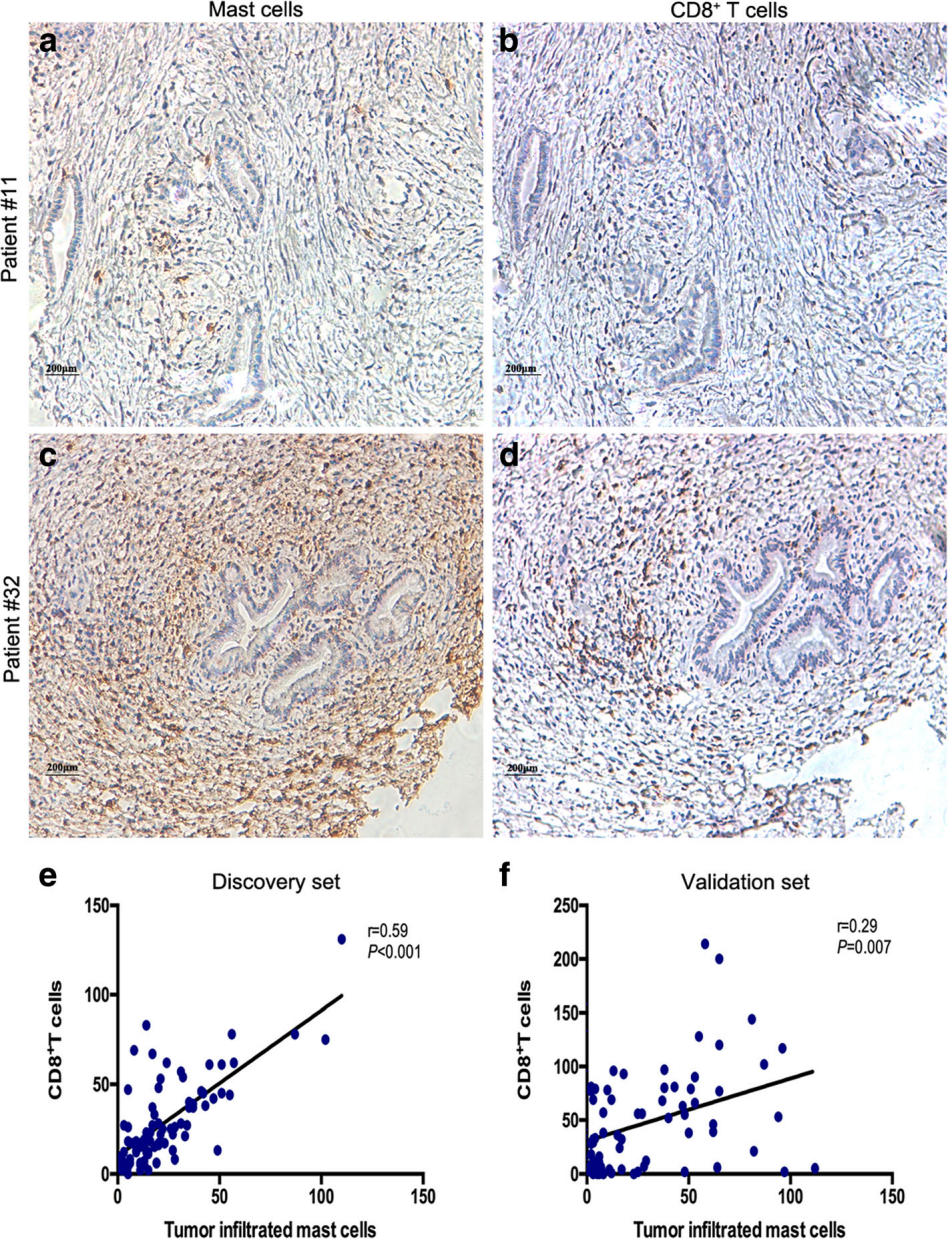
The relationships between TIMs and CD8+ T cells in BTC patients. (**a**-**d**) serial sections from GBC and EBDC samples immunohistochemically stained for TIMs and CD8+ T cells. Scale bar: 200 μm (original magnification 200×). (**e**, **f**) Spearman’s correlation for TIMs and CD8+ T cells in the discovery set and validation set

Previous study about inflammation reported that activated mast cells induce CD8+ T cells through production of leukotrienes [26]. Considering that TIMs predict favorable prognosis in this study, it was postulated that TIMs may inhibit tumor progression depend on the anti-tumor effect of CD8+ T cells. Therefore, we detected and analyzed the infiltration of CD8+ T cells in the tumor tissues. Interestingly, we found TIMs infiltration was positively correlated with CD8+ T cells in the discovery and validation set, respectively (Fig. 3, *r* = 0.59, *P* < 0.001; *r* = 0.29, *P* = 0.007). The associations between CD8+ T cells and overall survival in the discovery set and validation set are list in Additional file 2: Figure S1.

## Discussion

A large number of studies have tried to identify the contributory functions of tumor associated mast cells in tumor growth [16]. In the studies about colon, [27] gastric, [28] pancreas, [29] bladder cancers, [12] mast cells appear to be pro-tumorigenic and are associated with poor prognosis. However, Welsh et al. demonstrated that in non-small-cell lung cancer, tumor cell islet mast cells had a strong independent association with an improved prognosis and the stromal mast cells number was not significantly associated with survival [23]. In contrast to above studies, we observed that TIMs was mainly located in tumor stroma tissues and was positively correlated with prolonged overall survival. These results indicated that TIMs, which is modified by their environment, could display different phenotype and thus exert beneficial or detrimental effects on tumor progression. Previous study about hepatocellular carcinoma reported that peritumoral mast cells play a critical role in the suppression of immune reactions of tumors and cooperate with Tregs to sustain allograft tolerance and exacerbate tumor immunosuppression [30]. Nakae et al. demonstrated that mast cells activate T cells via TNFα release and through cell-cell interactions via OX40L [31]. Considering the role of mast cells plays in the developing tumor, we proposed that mast cells might present an anti-tumor effect through activating cytotoxic T cells and inducing an immune-stimulating environment in this study. Spearman’s correlation analysis indicated that TIMs stromal infiltration was positively correlated with anti-tumor CD8+ T cells. These findings confirmed the hypothesis that TIMs may play a critical role in activating and promoting CD8+ T cells to reject tumors. Several previous studies of inflammation suggested that activated mast cells facilitate anti-infection by enhance recruitment of NK and CD8+ T cells [31, 32]. Sharon A. Oldford et al demonstrated that mast cells could display antitumor activity by recruiting CD8+ T cells via secreting CCL3 in the tumor context [33]. The stromal infiltration of CD8+ T cells in tumor context is the basis for patients who receive immunotherapy. Accordingly, TIMs might serve as an important prognostic factor in identifying biliary tract cancer patients for the combination of chemotherapy and immunotherapy.

Gemcitabine-based ACT is an important modality for patients with advanced biliary tract cancer after surgery. More and more recent studies demonstrated that administration of gemcitabine or combined with platinum could significantly prolong survival of patients with advanced biliary tract cancer [34–36]. However, few researches focused on identifying patients whose tumor will be sensitive to ACT [7]. Therefore, we compared the OS of patients who did or not receive gemcitabine-based ACT and found that those who suffered from high TIMs stromal infiltration could significantly benefit from gemcitabine-based ACT. Considering TIMs in biliary tract cancer were positively associated with CD8+ T cells, it suggested that high TIMs infiltration correlated with an immune-stimulated microenvironment and inhibited tumor progression via recruitment of antitumor immune cells. These findings indicate that TIMs could be a vital factor for predicting chemotherapeutic response, which could be valuable for selection and management of patients who receive ACT. Therefore, it is essential to stratify that if patients could benefit from ACT and avoid the excessive toxicities of ACT.

The study has limitations that it is a retrospective single institution research and sample size is relatively small. Moreover, the results may not be generalizable to other populations and to patients treated with adjuvant capecitabine. In addition, the results were based on immunohistochemistry of tissue microarrays, which is a semiquantitative method and may not be typical. A prospective, larger, multi-centered randomized trial is required to validate these findings in future.

## Conclusions

In conclusion, TIMs infiltration in biliary tract cancer patients could predict favorable prognosis. Biliary cancer patients with high TIMs infiltration tend to have improved outcomes after receiving adjuvant gemcitabine-based ACT. High TIMs infiltration was correlated with more infiltration of CD8+ T cells, which may provide a pro-inflammatory context and a guidance for immunotherapy.

## Additional files

Additional file 1: **Table S1.** Association between CD8+ T cells infiltration and patient characteristics. (DOC 64 kb)

Additional file 2: **Figure S1.** Association between CD8+ T cells and overall survival in the discovery set and validation set. (A,C) Kaplan-Meier analysis of overall survival in the discovery set and validation set based on CD8+ T cells infiltration (B,D) Kaplan-Meier analysis of overall survival in the discovery set and validation set based on combination of TIMs and CD8+ T cells infiltration. (TIFF 652 kb)

## Abbreviations

ACT: Adjuvant chemotherapy treatment
BTC: Biliary tract cancer
EBDC: Extrahepatic bile duct cancer
GBC: Gallbladder cancer
MCP: Monocyte chemotactic protein
MMP: Matrix metalloproteinase
SCF: Stem cell factor
TGF-β: Transforming growth factor beta
TIMs: Tumor-infiltrating mast cells
TMA: Tissue microarray
