# Supplementary material for: Tumor-infiltrating mast cells predict prognosis and gemcitabine-based adjuvant chemotherapeutic benefit in biliary tract cancer patients
Source: BMC Cancer. 2018 Mar 21;18:313. doi: 10.1186/s12885-018-4220-1 (PMC5863450; doi:10.1186/s12885-018-4220-1)
Supplement: Supplementary file 1 — Table S1. Association between CD8+ T cells infiltration and patient characteristics. (DOC 64 kb) [file 12885_2018_4220_MOESM1_ESM.doc]

**Table S1.** **Association between CD8+ T cells infiltration and patient characteristics.**

|  |  | | | | | Discovery set (n=115) | | |  |  | | | Validation set (n=135) | | |
| --- | --- | --- | --- | --- | --- | --- | --- | --- | --- | --- | --- | --- | --- | --- | --- |
| Characteristic | Patients | | | | | CD8+ T cell infiltration | | |  | Patients | | | CD8+T cell infiltration | | |
|  | Number | | | % | | Low  (n = 53) | High  (n = 62) | *P*^a^ |  | Number | % | | Low  (n = 68) | High  (n = 67) | *P*^a^ |
| Age, years  Mean ± SD^b^ | 62.56 ± 10.26 | | | | | 63.75 ± 10.19 | 61.53 ± 10.28 | 0.25 |  | 63.67 ± 11.62 | | | 62.90 ± 10.94 | 64.69 ± 12.14 | 0.37 |
| Gender  Female  Male | 62  53 | | | | 53.91  46.09 | 28  25 | 34  28 | 0.85 |  | 91  44 | | 67.41  32.59 | 48  20 | 43  24 | 0.47 |
| Tumor location  Perihilar  Distal  Gallbladder | 40  12  63 | | | | 10.43 63  54.78 | 16  6  31 | 26  6  30 | 0.43 |  | 24  10  101 | | 17.78  7.41 74.81 | 13  4  50 | 11  6  51 | 0.75 |
| T-stage  T1-2  T3  T4 | 41  44  30 | | | | 35.65  38.26  26.09 | 17  19  17 | 24  25  13 | 0.40 |  | 16  79  40 | 11.85  58.52  29.63 | | 10  37  21 | 6  42  19 | 0.49 |
| N-stage  N0  N1,2 | 62  53 | | | | 53.91  46.09 | 27  26 | 35  27 | 0.58 |  | 114  21 | 84.44  15.56 | | 56  12 | 58  9 | 0.64 |
| TNM stage  I-II  III  IV | 39  36  40 | | | | 33.91  31.30  34.79 | 17  13  23 | 22  23  17 | 0.16 |  | 16  74  45 | 11.85  54.81  33.34 | | 10  33  25 | 6  41  20 | 0.30 |
| Differentiation  Well, Moderate  Poor | 76  39 | | | | 66.09  33.91 | 33  20 | 43  19 | 0.43 |  | 59  76 | 43.70  56.30 | | 27  41 | 32  35 | 0.39 |
| Residual tumor  R0  R1 | 100  15 | | 86.96  13.04 | | | 46  7 | 54  8 | 0.96 |  | 119  16 | 88.15  11.85 | | 64  4 | 55  12 | **0.036** |
| Vascular invasion  Absent  Present | 64  51 | 55.65  44.35 | | | | 28  27 | 36  24 | 0.35 |  | 106  29 | 78.52  21.48 | | 55  13 | 51  16 | 0.54 |
| ACT  Absent  Present | 65  50 | 56.52  43.48 | | | | 33  20 | 32  30 | 0.27 |  | 85  50 | 62.96  37.04 | | 45  23 | 40  27 | 0.48 |

SD = standard deviation; ACT=adjuvant chemotherapy;

a, *P*<0.05 is considered statistically significant.

b, The results of continuous variables are presented as mean±SD (standard deviation)­­­.
