# Supplementary figures and images for: Tumor-infiltrating mast cells predict prognosis and gemcitabine-based adjuvant chemotherapeutic benefit in biliary tract cancer patients
Source: BMC Cancer. 2018 Mar 21;18:313. doi: 10.1186/s12885-018-4220-1 (PMC5863450; doi:10.1186/s12885-018-4220-1)

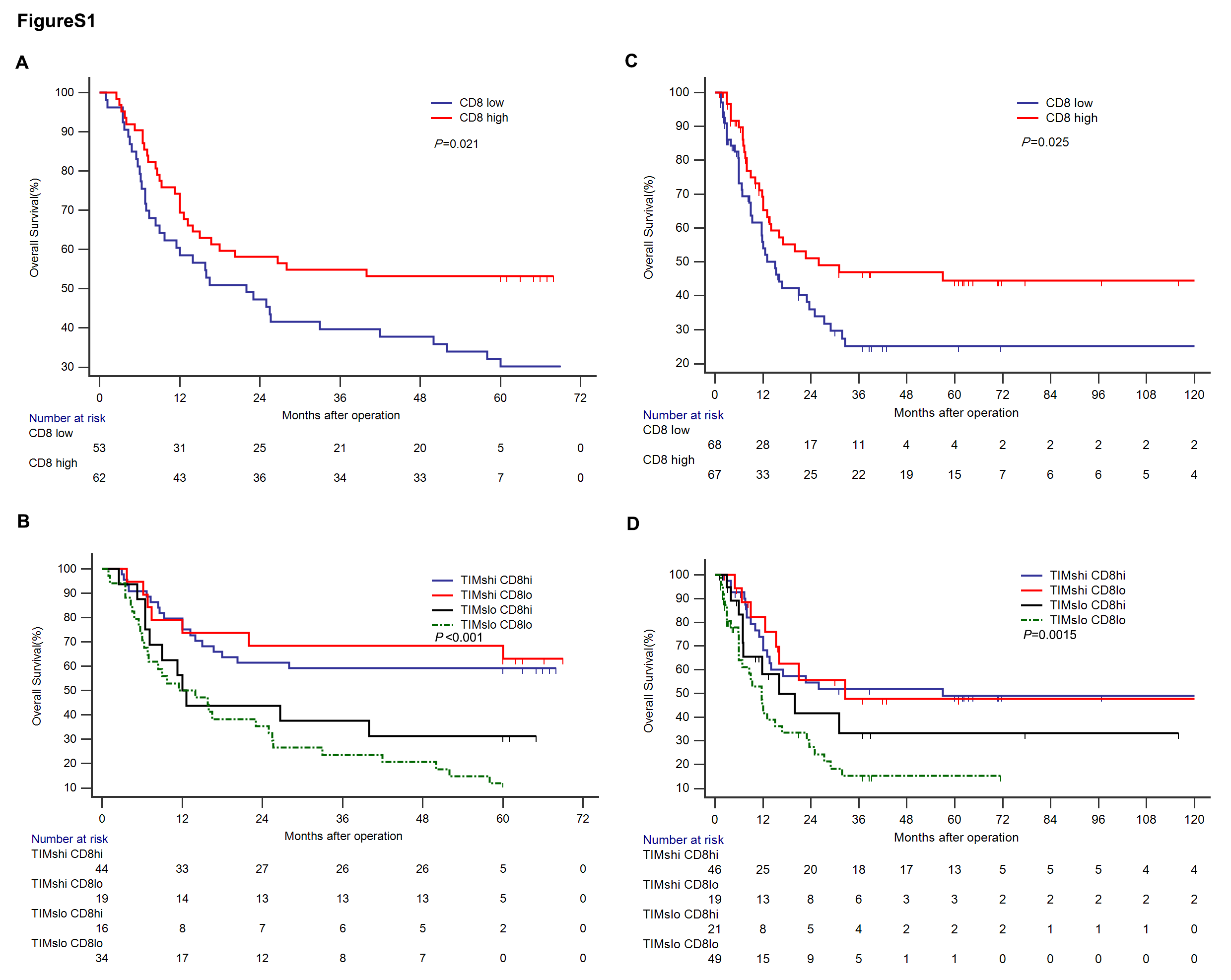

Supplement: Supplementary file 2 — Figure S1. Association between CD8+ T cells and overall survival in the discovery set and validation set. (A,C) Kaplan-Meier analysis of overall survival in the discovery set and validation set based on CD8+ T cells infiltration (B,D) Kaplan-Meier analysis of overall survival in the discovery set and validation set based on combination of TIMs and CD8+ T cells infiltration. (TIFF 652 kb) [file 12885_2018_4220_MOESM2_ESM.tif]
